# *Streptomyces fungicidicus*-derived secondary metabolites as an antiviral agent to alleviate zucchini yellow mosaic virus in squash

**DOI:** 10.1038/s41598-025-24821-y

**Published:** 2025-11-18

**Authors:** Said Behiry, Rokaia Nabil, Hosny Younes, Ahmed Heflish, Bassant Philip, Ahmed Abdelkhalek

**Affiliations:** 1 Agricultural Botany Department, Faculty of Agriculture (Saba Basha), Alexandria University, Alexandria, 21531 Egypt; 2 Plant Protection and Biomolecular Diagnosis Department, ALCRI, City of Scientific Research and Technological Applications, New Borg El-Arab City, 21934 Alexandria Egypt; 3 Plant Protection Department, The National Institute of Horticultural Research, Konstytucji 3 Maja 1/3, Skierniewice, 96-100 Poland

**Keywords:** *Streptomyces fungicidicus*, ZYMV, Defense system, polyphenolic compounds, GC-MS, Biotechnology, Microbiology, Plant sciences

## Abstract

Zucchini yellow mosaic virus (ZYMV) poses a significant threat to squash crops, causing severe symptoms and substantial yield losses. This study investigates the potential of *Streptomyces fungicidicus* as a biocontrol agent for managing ZYMV in squash plants by inducing systemic resistance. Approximately 95% of field-collected squash samples were positive for ZYMV, exhibiting chlorotic mottling, vein banding, and leaf distortion. The virus was isolated, purified, and confirmed through RT-PCR (Accession no. PV131044) and transmission electron microscopy (TEM), which displayed flexuous, filamentous particles typical of ZYMV. Among the isolated and tested *Streptomyces* spp., *Streptomyces fungicidicus* SF1-RSI2 (Accession no. PV489988) exhibited promising antiviral activity. GC–MS analysis of the SF1-RSI2 culture filtrate identified 35 bioactive compounds, with (–)-spathulenol being the most abundant (13.1%), followed by 9-octadecenoic acid methyl ester (9.24%) and triacetin (8.88%), suggesting a complex mixture of metabolites potentially contributing to the observed antiviral effects. Under greenhouse conditions, the foliar application of SF1-RSI2 culture filtrate, either as a pre-ZYMV-inoculation (protective) or post-ZYMV-inoculation (curative) treatment, enhanced plant growth, delayed symptom onset by up to 3 days, and reduced viral accumulation by up to 49.7% at 5 days post-inoculation (dpi) compared to untreated infected plants. The SF1-RSI2 applications significantly mitigated the detrimental effects of ZYMV on plant growth, chlorophyll content, and oxidative stress markers, such as H₂O₂ and malondialdehyde (MDA), while also notably increasing peroxidase enzyme activity and total phenolic content, indicating enhanced systemic resistance. Additionally, it influenced the transcriptional levels of defense-related genes, with protective treatment resulting in the highest expression levels of Cinnamate-4-hydroxylase (*C4H*), Cinnamate-3-hydroxylase (*C3H*), and Chalcone synthase (*CHS*), which are essential for plant defense mechanisms. HPLC analysis revealed a substantial increase in polyphenolic compounds, particularly chlorogenic acid, in plants treated with SF1-RSI2, highlighting its role in strengthening plant defenses. The findings suggest that *S. fungicidicus* promotes plant growth and enhances defense mechanisms, presenting a viable biocontrol strategy for managing viral infections in squash.

## Introduction

Plant diseases pose a significant threat to global food security and human well-being, resulting in substantial crop losses worldwide^1^. Among these pathogens, plant viruses are particularly substantial, leading to considerable crop production issues once they infect fields^2,3^. Squash (*Cucurbita pepo* L.) is a globally important vegetable crop, including in Egypt^4,5^, but its productivity is severely challenged by viral diseases, particularly zucchini yellow mosaic virus (ZYMV). ZYMV, a single-stranded positive-sense RNA virus (genus *potyvirus*; family *potyviridae*), is encapsulated in flexible filamentous particles measuring 750 nm in length and 12 nm in diameter, with a genome of 9,600 nucleotides^6^. Infected plants exhibit severe mosaic symptoms, necrosis, and malformations^7^, along with yellowing, stunting, and severe leaf and fruit deformities, which can potentially reduce yields by up to 94%^8^. Similar to other *Potyviruses*, ZYMV is transmitted non-persistently by several aphid species, making its management particularly challenging^9^.

Given the significant economic importance of squash crops, substantial efforts have been dedicated to controlling ZYMV through cross-protection and the development of new resistant squash varieties. Plant viral diseases can be managed using physical, chemical, and biological methods^10^. Physically, ZYMV spread can be mitigated using several approaches, including reflective or polyethylene mulches and floating row covers, which effectively deter aphid vectors and reduce virus transmission, as demonstrated in squash and other cucurbits^11,12^. Trap or barrier crops, such as maize, can be planted around squash to divert aphids away from the main crop. Planting delay or adjustment of sowing dates can also reduce infection by avoiding peak aphid activity periods. Moreover, roguing, or physically removing and destroying infected plants, helps lower the overall viral load in the field^13,14^. Chemical control strategies primarily rely on insecticides to suppress aphid populations and limit virus spread. However, their efficacy is often limited due to the non-persistent transmission nature of the virus^15^. The biological control strategy, which this study focuses on, uses beneficial microbial agents that have drawn considerable attention in the treatment of viral plant diseases because they provide a friendly and safe means of virus control^16,17^. Many biocontrol agents used for controlling viral plant diseases belong to the genus *Streptomyces*, a large group of actinobacteria comprising over 780 species and 30 subspecies^18^. *Streptomyces* species have demonstrated their value in the biocontrol of bacterial and fungal diseases in plants by disrupting the interactions between plants and pathogens^19^. Many researchers have revealed that the use of *Streptomyces* spp. to control plant viruses is limited, and the mechanism by which they might function as antiviral agents remains unknown^20–22^.

The potential of *Streptomyces* as biological control agents is primarily attributed to their ability to produce a diverse range of bioactive metabolites^23^. Recent studies have highlighted the antiviral potential of *Streptomyces*-derived metabolites. For instance, Taha et al.^24^ reported that culture filtrate from *Streptomyces ovatisporus* LC597360 achieved a 93.9% reduction in Tomato mosaic virus (ToMV) symptoms and viral load in tomato plants while enhancing the activities of antioxidant enzymes and promoting plant growth. Similarly, Ghanem et al.^20^ demonstrated that culture broth from *S. sampsonii*, *S. rochei*, and *S. griseus* significantly inhibited ZYMV replication in squash, induced systemic resistance, and improved growth, with behenic alcohol (docosanol) identified as a key metabolite associated with antiviral activity. In another study, Nasr-Eldin et al.^25^ showed that crude culture filtrate from *Streptomyces* spp. effectively protected potato plants against Potato virus Y (PVY^NTN^) by inducing systemic acquired resistance (SAR), resulting in reduced disease symptoms and lower viral titers. It was reported that some isolates (e.g., *S. cellulosae* Actino-48) reduce TMV lesion numbers and virus accumulation while inducing systemic resistance and upregulating defense enzymes in host plants^22^. Other strains act through secreted antiviral metabolites (e.g., metabolites from *S. ahygroscopicus* strain STZ, where the purified active compound was identified as ε-poly-L-lysine) or through extracellular polysaccharides (e.g., EPS66A from *Streptomyces* sp.), which prime host defenses and mitigate disease severity^26,27^. Meanwhile, *Streptomyces fungicidicus* is recognized as a potent biocontrol agent against gram-positive bacteria^28^; however, its antiviral activity has not yet been reported. Therefore, this study aimed to isolate, purify, and characterize the Egyptian ZYMV strain using standard methods, as well as to evaluate the most effective techniques for applying *Streptomyces fungicidicus* culture filtrate to squash plants to improve growth efficiency and resistance to ZYMV infection for the first time. To examine the mechanism by which resistance is induced, we evaluated the levels of antioxidant enzymes (POD), total protein, non-enzymatic oxidative stress markers (H_2_O_2_ and MDA), total phenolic content, and DPPH free radical scavenging activity. Furthermore, we examine the levels of expression of defense-related genes in squash, including chalcone synthase (*CHS*), cinnamate-4-hydroxylase (*C4H*), and *p*-coumarate 3-hydroxylase (*C3H*). Additionally, we investigated the predominant active compounds present in the metabolites of *S. fungicidicus* culture filterate and squash leaf extract using GC-MS and HPLC techniques.

## Materials and methods

### Plant materials

Squash plants (*Cucurbita pepo* L.) collected from open fields (latitude: 31.129936, longitude: 29.923781) in Alexandria Governorate, Egypt, were used for virus isolation, following permission from the local farm owners. The plant species utilized (*C. pepo* L.) is widely cultivated and is not recognized as threatened or endangered according to the IUCN Red List or CITES. All experimental and field collection procedures adhered to institutional, national, and international regulations and ethical standards.

### Virus isolation, molecular characterization, and construction of a phylogenetic tree

Squash plants exhibiting characteristic symptoms of ZYMV infection, such as leaf mosaic, chlorosis, and leaf deformation, were collected for assessment of viral infection. ELISA was performed using a polyclonal antiserum against ZYMV obtained from the German Collection of Microorganisms and Cell Cultures (DSMZ, Braunschweig, Germany) to detect viral infection in the samples. Briefly, ZYMV was isolated and purified using the single local lesion technique on *Chenopodium amaranticolor*, following the method described by Desbiez and Lecoq^29^. This method allowed us to obtain a purified ZYMV isolate, which was subsequently maintained and propagated on squash plants (*Cucurbita pepo* L.) for further molecular and biological analyses. In an insect-proof greenhouse, the ZYMV isolate was mechanically inoculated on the squash plants. After verifying positive ELISA samples by RT-PCR using specific primers as described previously^30^, the total RNA from the infected plants was extracted using the Plant RNA Mini Kit, following the manufacturer’s instructions (Bioline, cat. No. Bio-52040). ZYMV-coat protein (ZYMV-CP) gene primers were used to synthesise the first-strand cDNA, which was then subjected to PCR amplification (Table 1). Using the Maxima reverse transcriptase kit, one µg of RNA was converted to cDNA^31^. As previously described^30^, PCR reactions were performed using 2 µL of produced cDNA and ZYMV-CP gene-specific primers. The PCR process consisted of a 2 min initial denaturation at 95 °C, followed by 35 cycles of 1 min at 95 °C, 1 min at 54 °C, and 1 min at 72 °C. The last extension took place for 7 min at 72 °C. The PCR products were purified using a PCR clean-up column kit, separated on a 2% agarose gel, stained with Red Safe, and examined using a gel documentation system. They were then sequenced using an ABI PRISM model 310 DNA sequencer. The annotated nucleotide sequence was compared to sequences of previously published ZYMV isolates using NCBI-BLAST (http://blast.ncbi.nlm.nih.gov/Blast.cgi). Then, the sequence was assigned an accession number and deposited in the GenBank database. To create a phylogenetic tree, several sequence alignments were compared using MEGA 11 ^32^.

**Table 1:** Nucleotide sequences of primers used in this study.

Gene name	Direction	Nucleotide sequences (5’-….-3’)	References
Zucchini yellow mosaic virus-coat protein (*ZYMV-CP*)	Forward	GGACAGTGCGACTATAGCTTCAA	^33^
	Reverse	TTTAACCGCGAATTGCGTATC	
16 S ribosomal RNA (16 S rRNA)	Forward	GAAGAGTTTGATCCTGGCTCAG	^34^
	Reverse	CTACGGCTACCTTGTTACGA	
Chalcone synthase (*CHS*)	Forward	ACGGACATTTGAGGGAAGTG	^35^
	Reverse	ACCTAGTTTCGCCTCCACCT	
Cinnamate-4-hydroxylase (*C4H*)	Forward	ACATCAATGTGGCAGCGATA	^35^
	Reverse	GAAACCAACTTGGCAACGAT	
*p*-Coumarate 3-hydroxylase (C3H)	Forward	TTGGTGGCTACGACATTCCTAAGG	^30^
	Reverse	GGTCTGAACTCCAATGGGTTATTCC	
Elongation factor 1-alpha (*EF1a*)	Forward	GCTTGGGTGCTCGACAAACT	^36^
	Reverse	TCCACAGAGCAATGTCAATGG	
Beta-actin (*β*-*actin*)	Forward	TGGACTCTGGTGATGGTGTTA	^37^
	Reverse	CAATGAGGGATGGCTGGAAAA	

### Purification of ZYMV and transmission electron microscopy analysis

The purified ZYMV isolate was prepared as previously described^38^, with some modifications. Briefly, 100 g of fresh, systemically infected squash leaves, collected three weeks after inoculation, were mixed with 300 mL of extraction buffer (comprising 0.5 M K_2_HPO_4_, 0.02 M Na_2_SO_3_, 0.01 M NaS_2_CN(C_2_H_5_)_2_, and 0.005 M C_10_H_16_N_2_O_8_, pH 8.5) and homogenized with a blender. The homogenate was filtered using three layers of cheesecloth, followed by the addition of 3% Triton X-100 (v/v). The mixture was subsequently subjected to a gradual addition of 25% CHCl_3_ and 25% CCl_4_ (v/v), and stirred for 30 min. Centrifugation was conducted at 12,000 rpm for 15 min at 4 °C. In the aqueous phase, 8% polyethylene glycol 6000 and 3% NaCl (w/v) were added, and the solution was stirred for 1 h at 4 °C. The solution was then centrifuged at 12,000 rpm for 20 min at 4 °C. The pellets were resuspended in 0.05 M sodium citrate buffer at pH 7.5 and incubated at 4 °C overnight with periodic stirring. The suspension was subjected to centrifugation at 12,000 rpm for 20 min at 4 °C. The supernatant was subjected to high-speed centrifugation at 23,500 rpm for 2 h. The final pellet was resuspended in 0.05 M Na_3_C_6_H_5_O_7_ buffer at pH 7.5 and subjected to centrifugation at 9,000 rpm for 20 min. The purified virus concentration was assessed using UV/VIS spectrophotometry (PerkinElmer Lambda 25, PerkinElmer, Waltham, MA, USA) with an extinction coefficient of 5 mg/mL cm-1 at 260 nm. For transmission electron microscopy (TEM), formvar-coated nickel grids were placed on drops of the purified virus preparation for 5 min, rinsed with distilled water, stained with 2% H_3_PW_12_O_40_ (pH 7.0), and analyzed using a TEM (Hitachi HT7700, Hitachi High-Tech, Tokyo, Japan)^39^.

### *Streptomyces* isolation, characterization, and molecular identification

Soil samples were collected from various rhizosphere soils in the Egyptian governorate of Alexandria (31.141508, 29.966555) between 11 and 15 cm below the surface and stored in sterile plastic bags. Once in the laboratory, 1 g of each collected sample was combined with 9 mL of regular saline, vortexed, and then serially diluted (1 × 10^−1^, 1 × 10^−2^, and 1 × 10^−3^) in a set of test tubes. Then, 0.1 mL of the sample from each dilution tube was transferred to International Streptomyces Project medium 4 (ISP-4 agar medium) as previously described^40^, distributed using a sterile glass rod, and incubated at 37 °C for 7 days. Additionally, the cultures were purified using the streak plate procedure. To prepare the *Streptomyces* culture filtrate (CF), pure colonies of each isolate were inoculated into ISP-4 broth medium and incubated at 30 °C for seven days with shaking at 200 rpm. After incubation, the cultures were filtered through two layers of filter paper (8–10 μm pore size) to remove mycelial biomass. The resulting filtrate was centrifuged at 10,000 rpm for 10 min to eliminate any remaining particulate matter, and the supernatant was subsequently sterilized using a 0.22 μm Millipore filter. The sterile CF was then used for all subsequent antiviral assays, ensuring that only the metabolites and soluble compounds produced by the *Streptomyces* isolates were applied, not the bacterial cells themselves. The antiviral efficacy of CF was assessed on *Nicotiana glutinosa* using the half-leaf technique^41^. The isolate exhibiting the highest percentage of inhibition was selected for morphological and molecular identification and subsequently chosen for further experimentation. The promising *Streptomyces* isolate was cultured in 150 mL conical flasks with ISP-4 broth for 5–7 days. The mycelia were harvested from the broth by centrifugation at 10,000 rpm for 3 min, and the DNA was isolated using a conventional phenol-chloroform extraction process^42^. A thermal cycler (Techne Prime, Cambridge, UK) was used to assess the amplification of the 16 S rRNA gene using P0 and P6 primers (Table 1), as previously described^43^. Using NCBI-BLAST, the annotated nucleotide sequence was matched to sequences of similar isolates that had already been published. The sequence was then submitted to GenBank to obtain an accession number. Phylogenetic trees were constructed by comparing several sequence alignments using MEGA 11^32^.

### Greenhouse experimentation and sampling

Virus-free seeds of the new Eskandrany H1 cultivar of the squash (*Cucurbita pepo* L.) plant were obtained from the Ministry of Agriculture, Agricultural Research Center, Egypt. In a greenhouse experiment, the effect of *Streptomyces*-CF on the induction of resistance in squash plants against ZYMV was investigated. The experiment was conducted under conditions of 28 °C for 16 h /16°C for 8 h (day/night) with a relative humidity of 70%. The squash seeds were grown in plastic pots (20 cm in diameter) containing a 3 kg pre-sterilized soil mixture of clay, sand, and peat moss, prepared in a 1:1:1 ratio. The seeds were sown in the pots, and after 7 days, similar seedling was selected to start the experiment. Five treatment groups were established to evaluate the effects of *Streptomyces*-CF on squash plants (Table 2). Each treatment included five biological replicates, with five plants per pot. During the foliar spraying treatments, each plant received approximately 20 mL of the prepared solution using a handheld atomizer, ensuring uniform coverage of the entire foliage until runoff. All plant groups were kept in a greenhouse for two weeks, and each day was checked for the emergence of symptoms. Squash leaves were picked from younger leaves, and the collection process was repeated two times, 5 and 10 days post-viral inoculation (dpi). Ten days after ZYMV inoculation, squash plants from each group were harvested, rinsed multiple times, and then their fresh and dry weights (g) were measured. HPLC analysis was then performed. Five squash plants, with three leaves per plant in each pot, yielded a pool of fifteen leaves for each treatment, which served as the independent biological replication for additional study. Every biological replicate was subjected to three technical replications to ensure an accurate assessment. The indirect ELISA test was used to estimate the ZYMV accumulation level. The growth parameters, enzyme activity, protein content, total phenolic compounds, DPPH free radical scavenging activity, and non-enzymatic oxidative stress markers (H_2_O_2_ and MDA) were all recorded at two different time intervals at 5 and 10 dpi. The plants were also used to record the effect of bioagent applications on the following growth parameters: shoot length (cm), root length (cm), shoot and root fresh weight (g), shoot and root dry weight (g), and chlorophyll content (SPAD unit).

**Table 2:** Description of treatment groups used to evaluate the effect of *Streptomyces* culture filtrate (CF) on squash plants inoculated with ZYMV.

Treatment code	Description	Purpose
Mock	Plants were sprayed with sterile ISP-4 broth and mechanically inoculated with buffer only.	Negative control (healthy plants)
ZYMV	Plants were mechanically inoculated with ZYMV and sprayed with sterile ISP-4 broth.	Positive control (infected, untreated plants)
*S. fungicidicus*	Plants were sprayed with *Streptomyces*-CF and mechanically inoculated with buffer.	CF effect without virus
Pre-ZYMV	Plants were sprayed with *Streptomyces*-CF 24 h before ZYMV inoculation.	Protective/induced resistance effect
Post-ZYMV	Plants inoculated with ZYMV and sprayed with *Streptomyces*-CF 24 h after viral inoculation.	Curative effect

### Determination of peroxidase activity and total protein content

After being powdered in liquid nitrogen, 1 g of the leaves was combined with 3 mL of potassium phosphate (50 mM, pH 7.5). After that, the mixture was centrifuged for 30 min at 4 °C and 12,000 rpm. The protein content was determined, and peroxidase activity was measured using the resultant supernatants. The formula for the activity of the peroxidase (POD) was stated by Rached-Kanouni and Alatou^44^. By mixing 1.5 mL of 0.1 M phosphate buffer (pH 6.8) containing 30 mM H_2_O_2_ and 30 mM guaiacol with 0.15 mL of enzyme extract, the optical density (OD) at 470 nm was measured every 20 s. A unit of peroxidase activity was expressed in mM/g FW, as the change in absorbance per minute, and as specific activity, defined as enzyme units per mg of soluble protein. Bradford’s formula for total protein was used^45^. Initially, 50 µL of extract, 2.5 mL of Coomassie blue, and a 0.15 M NaCl solution were utilized as solvents. A 50 µL of 0.15 M NaCl and Coomassie blue were employed as blanks. The reaction mixtures were allowed to reach room temperature for 5 min. Bovine serum albumin was used to create standard solutions in a range of concentrations (1–10 µg/mL). After measuring the absorbance at 595 nm against a blank, the protein concentration of the extract was calculated using the standard curve.

### Estimation of oxidative stress markers

Two oxidative stress markers, hydrogen peroxide (H_2_O_2_) and malondialdehyde (MDA), were evaluated. MDA, a result of the peroxidation of unsaturated fatty acids, was quantified using the method described by Zhang and Kirkham^46^. A fresh leaf weighing 250 mg was homogenized in 5 mL of 0.1% (W/V) trichloroacetic acid (TCA) and centrifuged for 15 min at 6000 rpm. A 1 mL sample was heated to 95 °C for 30 min and then cooled in an ice bath. This was then mixed with 4 mL of 0.5% (w/v) thiobarbituric acid (TBA) in 20% (v/v) trichloroacetic acid (TCA) and spun. The absorbance at 532 nm and 600 nm, respectively, was measured in the supernatant using a Beckman spectrophotometer (DU730, Beckman Coulter Inc., Brea, CA, USA). A (155 mM^−1^ cm^−1^) absorption coefficient, a reading output of 532 nm subtracted from a reading output of 600 nm, and the expression nmol/g FW were used to calculate the MDA concentration. To determine the concentration of H_2_O_2_, 200 mg of tissue was ground into a powder in a pre-chilled mortar, according to Velikova^47^. The powdered sample was placed in a 2 mL tube with 0.1% w/v of cooled TCA, homogenized with a vortex, and centrifuged at 10,000 rpm for 15 min. Each well should contain 50 µL of reaction mixture, 50 µL of supernatant, 50 µL of 10 mM potassium phosphate buffer (pH 7.0), and 100 µL of 1 M KI. Calculate the H_2_O_2_ concentration by reading the absorbance at 390 nm using a standard curve with known H_2_O_2_ values. Using known H_2_O_2_ concentrations, draw a standard curve against its O.D. and use the formula y = mx + c, where m = y2-y1/x2 -x1, to get the sample concentration. The concentration of H_2_O_2_ was reported in micromoles per gram of fresh weight (µmol/g FW). Formula for determining H_2_O_2_: H_2_O_2_ (µM/g FW) = C x Vt/Vr x W, where C represents content as calculated from the standard curve; Vt is the amount of extraction solution needed to pulverize the sample; Vr is the reaction’s supernatant volume; W = weight of leaf sample.

### Total phenolic content (TPC) and DPPH Estimation

One gram of dried leaves was extracted with 10 mL of 96% methanol using shaking at 200 rpm for 2 h, followed by centrifugation at 10,000 rpm to obtain a clear extract solution. Using the Folin-Ciocalteu reagent, the TPC was measured according to Velioglu et al.^48^, with slight modifications. Briefly, 900 µL of distilled water, 0.5 mL of 2 N Folin-Ciocalteu reagent, 100 µL of sample, and 2.5 mL of a 20% sodium carbonate (Na_2_CO_3_) solution were added. The absorbance against the prepared blank was measured at 725 nm following a 20-min incubation period at 23 °C. Using a calibration curve with gallic acid, the TPC was expressed as mg of gallic acid equivalents per gram of dry matter (mg of GAE/g of DM). The calibration curve’s range was 0.05–0.15 mg/mL (R2 = 0.99). On the other hand, the DPPH was assessed according to Philippe et al.^49^. The radical-scavenging activity of DPPH was used to evaluate the antioxidant activity of the plant extracts. To initiate the antioxidant reaction, 0.5 mL of plant extract and 3.5 mL of a freshly prepared DPPH methanol solution (containing 4 mg DPPH in 100 mL of methanol) were added to a tube. After 30 min of dark incubation at room temperature, absorbance at 517 nm was measured using a UV-visible spectrophotometer. The extract’s DPPH inhibition percentage (I%) was calculated using the following formula: I% = (A0-AT/A0) X 100, where A0 is the absorbance of the control (methanol-water with DPPH) and AT is the absorbance of the sample.

### Quantitative real-time PCR (qRT-PCR) analysis of ZYMV and defense-related genes

The total RNA was extracted from 100 mg of fresh squash leaves collected at 5 and 10 dpi using the guanidium isothiocyanate extraction procedure with some modifications^50^. Using a Nano SPECTROstar spectrophotometer, the concentration and purity of the extracted RNA were ascertained. A total of 20 µL of RT-PCR reaction mixture was made up of 1 µL of DNase-treated total RNA (100 ng/µL), 2 µL of 2 mM dNTPs, 5 µL of random hexamer primers, 0.3 µL of Easyscript RT reverse transcriptase enzyme, 4 µL of Easyscript RT buffer, and 7.7 µL of molecular-grade water. After 1 h of incubation at 42 °C, the reverse transcription process was inactivated by heating for 7 min at 80 °C. The reaction mixture was maintained at 4 °C and then stored at -20 °C until use. The transcriptional levels of three defense-related genes, *CHS*, *C4H*, and *C3H* (Table 1), were evaluated at the two time intervals using the qRT-PCR technique^51^. The two housekeeping genes, *EF1α* and *β-actin* (Table 1), were used as reference genes for normalizing the expression levels of different genes. A triple analysis was performed on each sample. In a real-time PCR, we performed 40 cycles of the thermal program, consisting of 94 °C for 5 s, 56 °C for 50 s, and 72 °C for 30 s, using a QIAGEN Rotor-Gene instrument. Through melting curve analysis, the specificity of the PCR amplicons was verified. The relative expression ratio was measured correctly and computed using the 2^−∆∆Ct^ technique^52^.

### Preparation of ethanol extract and HPLC analysis conditions

In a shaker, 2 g of the air-dried squash leaves from each treatment were macerated and extracted with 15 mL of 96% ethanol for 5 h. The cleared supernatant was obtained after filtration and centrifugation at 10,000 rpm for 20 min. It was then evaporated at 30 °C and stored in a dark tube. The phenolic and flavonoid-type compounds were identified using an Agilent 1260 Infinity HPLC series, equipped with a quaternary pump and a Zorbax Eclipse Plus C18 column (100 mm × 4.6 mm i.d.) (Agilent Technologies, CA, USA), operated at 30 °C^53^. HPLC-grade water, 0.2% phosphoric acid, acetonitrile, and methanol were mixed to form the mobile phase. The VWD detector was calibrated at 284 nm, and the injection volume was 20 µL. Many of the standard polyphenolic compounds used came from Merck KGaA in Darmstadt, Germany. They were methyl gallate, caffeic acid, syringic acid, pyrocatechol, rutin, ellagic acid, coumaric acid, vanillin, ferulic acid, naringenin, daidzein, quercetin, cinnamic acid, apigenin, kaempferol, and hesperidin.

### GC–MS analysis for identification of bacterial secondary metabolites

After being cultured for seven days, the *Streptomyces* culture broth precipitated, and the collected supernatant was combined with ethyl acetate as a solvent in a 1:1 (v/v) ratio to determine the active components of the *Streptomyces*-CF. The ethyl acetate phase and aqueous phase were separated using a separating funnel after the mixture was violently agitated for 20 min. In a rotary evaporator, the ethyl acetate extract was concentrated by evaporating it at 50 °C. GC-MS was used to analyze the residue, which comprised chemical components of secondary metabolites. The test was conducted using a GC-MS instrument (TRACE 1300 Series, Thermo, USA) for the analysis. Helium gas was used as a carrier at a flow rate of 1 mM/min, whereas the mass detector was operated in split mode. The mass range was 50–650 amu, the ramp rate was 4 min to 250 °C, and the injector was run at 250 °C. The oven temperature was initially set to 60 °C for 2 min, with a scan duration of 0.2 s. Mass spectra were obtained throughout a 53-min runtime at 70 eV. Following a comparison with data available in the GC-MS library in the literature, the components were identified^54^.

### Statistical analysis

Using the CoStat program, a one-way ANOVA was used to analyze the relative expression levels of five replicates for each set. A column bar shows the standard deviation (± SD), and the least significant differences (LSD) at a *p* ≤ 0.05 level of probability were used to identify significant differences between the relative expression levels. Relative expression numbers greater than 1 indicate up-regulation (an increase in gene accumulation) as compared to the control, while values less than 1 indicate down-regulation (a drop in expression levels).

## Results

### Virus isolation, purification, and molecular characterization

**Fig. 1:**
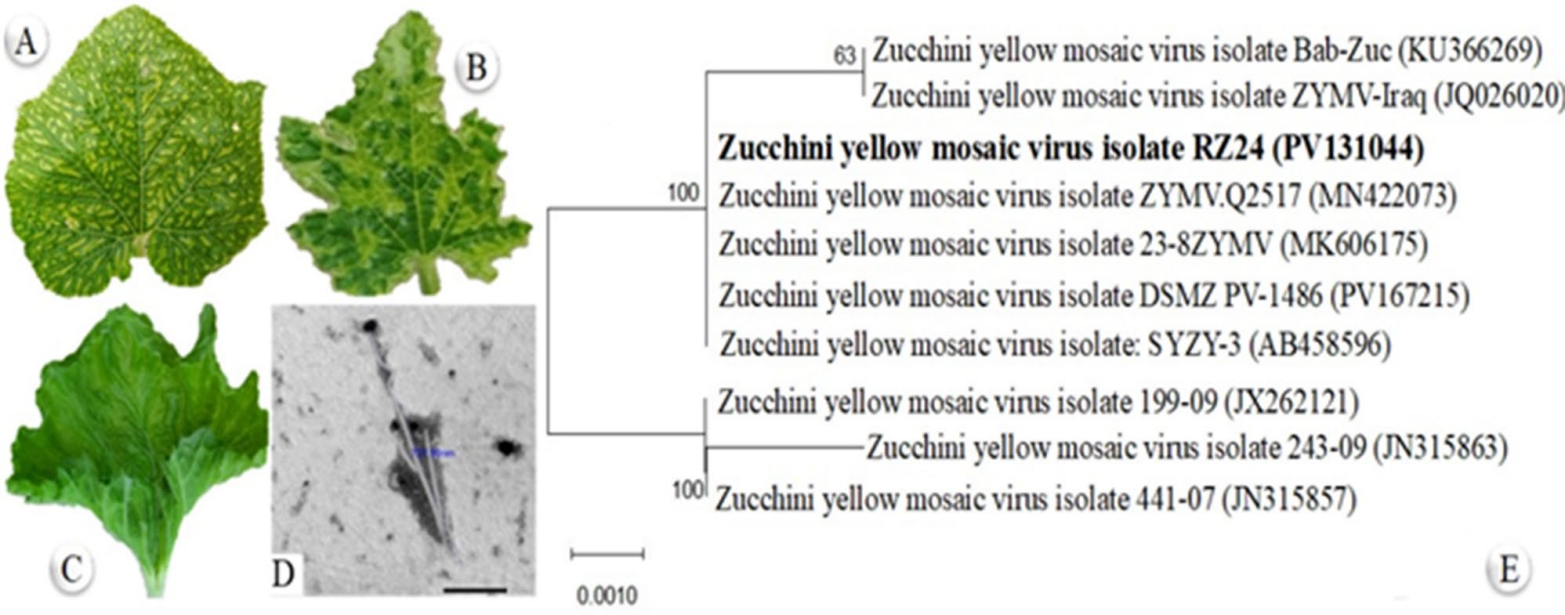
Characterization of Zucchini yellow mosaic virus (ZYMV) isolate RZ24 from infected squash plants. (**A**–**C**) Squash leaf exhibiting severe mosaic symptoms, including chlorotic mottling (**A**), vein banding (**B**), and blistering with leaf distortion (**C**). (**D**) Transmission electron microscopy (TEM) image showing flexuous, filamentous viral particles (~ 750 nm in length). Scale bar = 200 nm. (**E**) Phylogenetic tree based on coat protein (CP) gene sequences of ZYMV isolates, highlighting the RZ24 isolate (bold) clustering with reference isolates from GenBank. Bootstrap values (2000 replicates) are shown at nodes; the scale bar indicates nucleotide substitutions per site.

A total of 50 symptomatic squash plants (from approximately 150 leaves) were collected from open-field sites. Indirect ELISA revealed that approximately 95% of these samples were positive for ZYMV. Compared to healthy plants, ZYMV-infected plants exhibited a wide range of mild to severe symptoms, including chlorotic mottling, vein banding, blistering, and squash leaf distortion (Fig. 1A–C). After purification, the virus preparation yielded approximately 2.36 mg per 100 g of fresh infected tissue. TEM revealed that the purified ZYMV particles were flexuous, filamentous, and approximately 750 nm in length (Fig. 1D). Using RT-PCR with specific primers of *ZYMV-CP* gene, a 458-bp amplicon was successfully amplified from the infected tissue. The PCR product was subsequently purified, sequenced, and the resulting sequence was submitted to GenBank under accession number PV131044. The phylogenetic analysis of ZYMV isolate RZ24 revealed its genetic relationships with previously reported ZYMV strains (Fig. 1E). In the constructed tree, RZ24 (highlighted in bold) clusters closely with several reference isolates, including ZYMV.Q2517 (MN422073), 23-8ZYMV (MK606175), DSMZ PV-1486 (PV167215), and SYZY-3 (AB458596). The robustness of this clustering is supported by a bootstrap value of 100 for the branch containing RZ24 and its closest relatives. A high bootstrap value reflects strong statistical confidence in the inferred evolutionary relationships, confirming the reliability of the observed genetic associations. In contrast, some isolates, including Bab-Zuc (KU366269) and ZYMV-Iraq (JQ026020), are located on a separate branch with a lower bootstrap value of 63. This indicates that these isolates are more genetically divergent from RZ24, highlighting variation among global ZYMV strains. The evolutionary distances represented in the tree are relatively small, as indicated by the scale bar of 0.0010 substitutions per site (Fig. 1E). This suggests minimal genetic divergence among the closely clustered isolates, emphasizing the high sequence conservation of the CP gene within these ZYMV isolates. The phylogenetic analysis confirms that our RZ24 isolate is a typical representative of the virus, sharing a close genetic relationship with multiple geographically diverse isolates (Fig. 1E). The clustering pattern and high bootstrap support underscore the evolutionary conservation of the coat protein, providing insight into the genetic stability and widespread distribution of ZYMV strains.

### *Streptomyces* isolation and molecular characterization

**Fig. 2:**
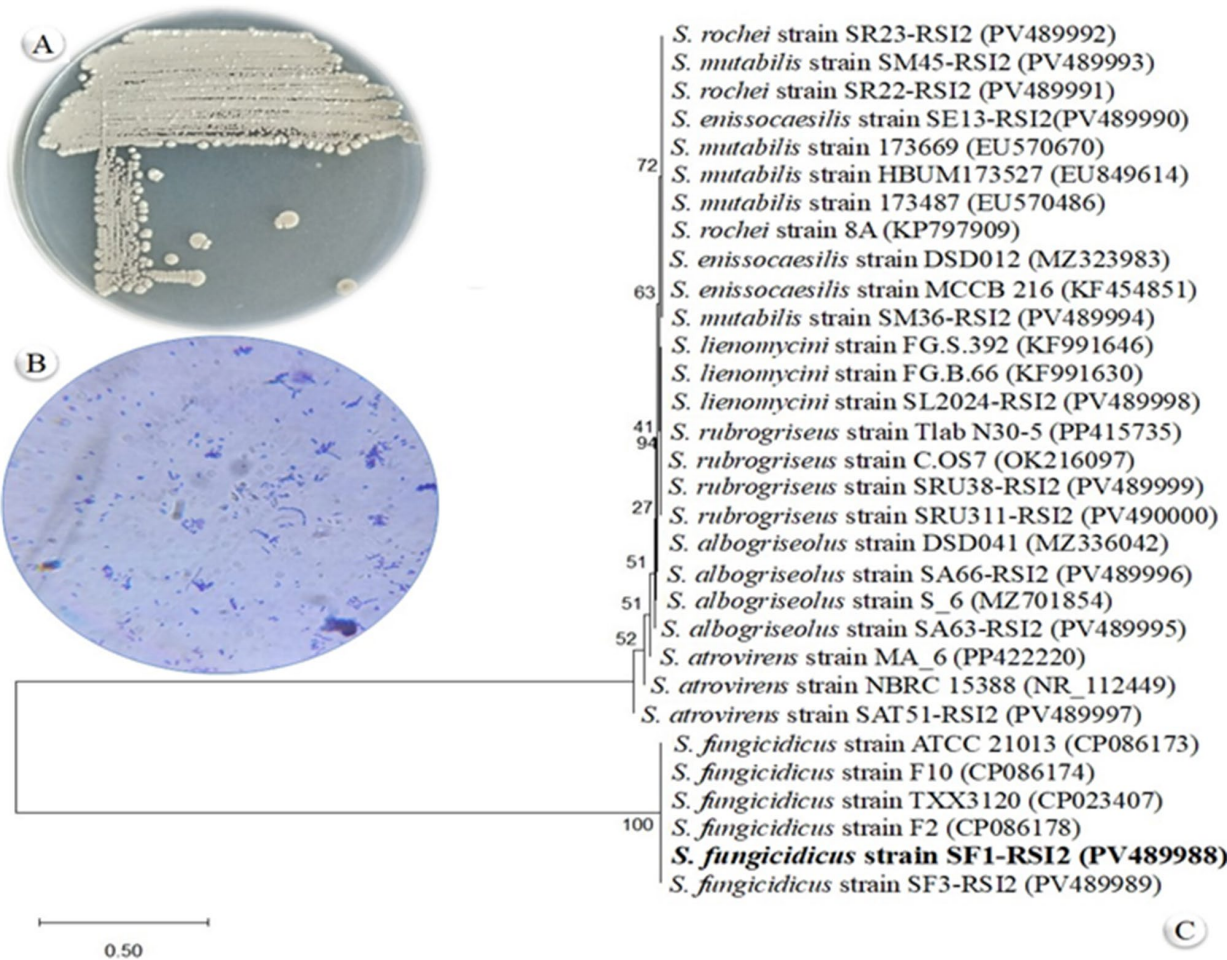
Morphological, microscopic, and phylogenetic characterization of actinomycete strain SF1-RSI2. (**A**) Colony morphology of SF1-RSI2 on ISP4 agar medium showing grey, circular colonies with irregular margins. (**B**) Microscopic observation of SF1-RSI2 after Gram staining, revealing filamentous, branched mycelia and spore-like structures. (**C**) Phylogenetic tree based on 16 S rRNA gene sequences showing the relationship of *Streptomyces fungicidicus* SF1-RSI2 (bold) with related *Streptomyces* species. Bootstrap values (expressed as percentages of 2000 replications) are shown at branch points. The scale bar represents 0.50 substitutions per site.

The morphological analysis of the isolated actinomycete revealed typical characteristics associated with the *Streptomyces* genus. The organism developed a well-branched, filamentous mycelium, producing both substrate and aerial structures. The aerial mycelium differentiated into chains of small, round to oval spores, often forming straight or slightly spiral chains. The colonies exhibited a leathery appearance with a powdery texture, resulting from the production of spores. The aerial mycelium was predominantly white, while the substrate mycelium was grey on ISP-4 medium (Fig. 2A). Gram staining confirmed the organism as a Gram-positive bacterium (Fig. 2B). Optimal growth was observed at 37 °C. Using NCBI-BLAST, the 1,500 bp sequence of the *16 S rRNA* from the isolated *Streptomyces* was compared to existing 16 S rRNA gene sequences of microorganisms in the NCBI GenBank. The isolated bacterial isolate was identified as *Streptomyces fungicidicus* and assigned the accession number PV489988 (SF1-RSI2). The 16 S rRNA gene sequence of strain SF1-RSI2 was compared with reference sequences from the GenBank database to determine its taxonomic position. In the phylogenetic tree (Fig. 2C), SF1-RSI2 (highlighted in bold) clusters tightly with other *S. fungicidicus* strains, indicating a close genetic relationship. This grouping is supported by a bootstrap value of 100, demonstrating very high confidence in the placement of SF1-RSI2 within the *S. fungicidicus* clade. Other *Streptomyces* species, such as *S. atrovirens*, *S. albogriseolus*, *S. rubrogriseus*, and *S. lienomycin*, are placed on distinct branches with lower bootstrap values, reflecting greater evolutionary distance from SF1-RSI2. The scale bar (0.50 substitutions per site) indicates the relative genetic divergence among the isolates. Overall, phylogenetic analysis confirms that SF1-RSI2 belongs to the *S. fungicidicus* species, consistent with its morphological and microscopic characteristics.

### Effect of *S. fungicidicus* on the symptom appearance, ZYMV accumulation, growth parameters, and total chlorophyll content

**Fig. 3:**
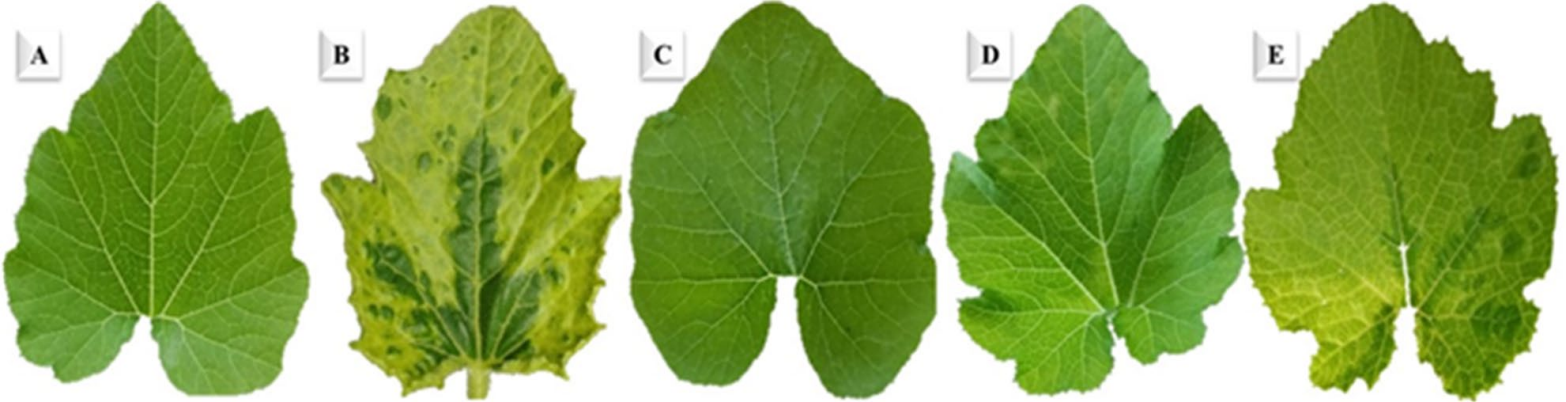
Disease symptoms on squash leaves infected with ZYMV at 10 dpi. (**A**) Mock-treated plants; (**B**) plants inoculated with ZYMV only; (**C**) Plants treated with *S. fungicidicus* only; (**D**) plants treated with *S. fungicidicus* 24 h before inoculation with ZYMV, and (**E**) plants treated with *S. fungicidicus* 24 h after inoculation with ZYMV.

**Fig. 4:**
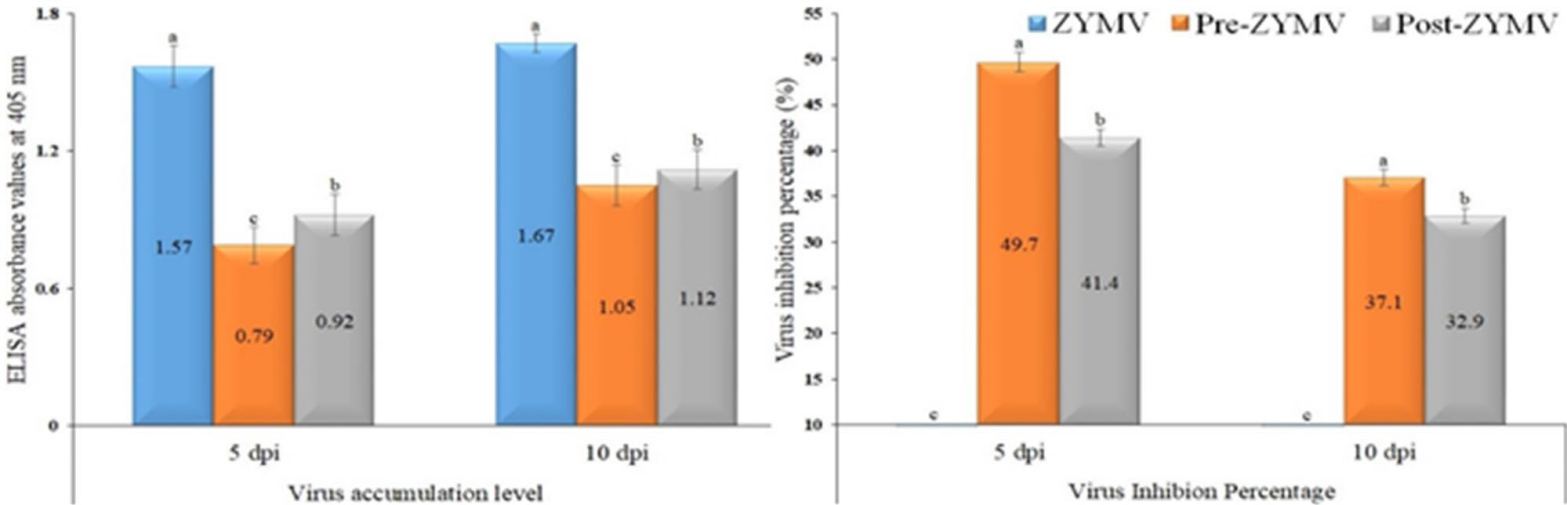
Accumulation levels and inhibition percentages of ZYMV in squash leaves at 5 and 10 dpi. Treatments include plants inoculated with ZYMV only (ZYMV), plants treated with *S. fungicidicus* 24 h before ZYMV inoculation (Pre-ZYMV), and plants treated with *S. fungicidicus* 24 h after ZYMV inoculation (Post-ZYMV). Columns sharing the same letter are not significantly different from each other based on mean values.

Under greenhouse conditions, the ZYMV treatment started to exhibit viral characteristic symptoms at 7 dpi and was visible at 9 dpi (Fig. 3). The protective treatment (Pre-ZYMV) delayed the onset of symptoms by 3 days, as they began to appear at 10 dpi. Similarly, curative treatment (Post-ZYMV) delayed the appearance of symptoms by 2 days. Neither the *S. fungicidicus*-treated plants nor the mock treatment showed any symptoms (Fig. 3). The ELISA results indicated that the ZYMV treatment exhibited the highest levels of viral accumulation at 1.57, in contrast to Pre-ZYMV and Post-ZYMV values of 0.79 and 0.92, respectively (Fig. 4). The findings demonstrate that the protective treatment led to a 49.7% reduction in virus concentration, whereas the curative treatment resulted in a 41.4% decrease at 5 dpi. Values of 1.67, 1.05, and 1.12 were reported for ZYMV, protective, and curative treatments at 10 dpi. The data indicated that protective treatments led to a 37.1% reduction in ZYMV accumulation, while curative treatments resulted in a 32.9% decrease in ZYMV accumulation (Fig. 4).

At 10 dpi, measurements of the growth parameters (Table 3) revealed that the shoot and root fresh weight (3.63 and 0.16 g, respectively) of the ZYMV treatment group were significantly lower than that of the mock-treatment plants (4.39 and 0.37 g, respectively) by approximately 17.3% and 56.7%, respectively. The foliar spray of *S. fungicidicus*-CF mitigates the effects of ZYMV on plant growth and weight. The protective treatment increased plant growth by 27% and 71.9% for the protective shoot and root fresh weight, respectively, and by 22.7% and 62.7% for the curative shoot and root fresh weight, respectively. The protective treatment, however, was slightly more effective than the curative treatment. Furthermore, protection treatment demonstrated noteworthy elevations in the dry weights of the shoots and roots, exhibiting growth percentages of 40.7% and 66.7%, respectively. Additionally, the protective treatment significantly outperformed the ZYMV treatment in terms of shoot and root lengths. Similarly, the curative treatment had longer squash shoots and roots than the ZYMV treatment, which showed increases of 17.1% and 11.7%, respectively. Additionally, a direct analysis of the chlorophyll content in all treatments was performed on fresh leaf samples (Table 3). Compared to the mock treatment (31.3 SPAD units), the results show a significant decrease in chlorophyll content due to the ZYMV treatment (28.9 SPAD units), representing approximately a 9% decrease. Conversely, the chlorophyll content increased with protective and curative treatments (30.8 and 28.5 SPAD units, respectively). More importantly, compared to the curative treatment, the protective treatment increased plant growth and chlorophyll levels. This demonstrates the importance of using the CF from *S. fungicidicus* as a protective treatment before infection to promote better growth and development.

**Table 3:** Growth parameters and chlorophyll content of squash plants following ZYMV and *S. fungicidicus* treatments.

Treatment	Shoot length (cm)	Root length (cm)	Fresh weight (g)		Dry weight (g)		Chlorophyll content (SPAD)
			Shoot system	Root system	Shoot system	Root system	
Mock	28.3 ± 2.25 c	18.3 ± 2.02 c	4.39 ± 0.63 d	0.37 ± 0.11 c	0.47 ± 0.12 cd	0.12 ± 0.09 c	31.3 ± 0.87 b
ZYMV	23.3 ± 3.51 d	15.1 ± 2.65 e	3.63 ± 0.59 e	0.16 ± 0.07 d	0.45 ± 0.07 d	0.09 ± 0.03 d	26.9 ± 2.03 e
*S. fungicidicus*	32.2 ± 3.21 a	22.3 ± 1.51 a	6.20 ± 0.61 a	1.20 ± 0.16 a	0.87 ± 0.46 a	0.43 ± 0.32 a	36.4 ± 3.40 a
Pre-ZYMV	29.8 ± 2.47 b	21.1 ± 1.76 b	4.97 ± 0.45 b	0.57 ± 0.12 b	0.76 ± 0.22 b	0.18 ± 0.03 b	30.8 ± 3.49 c
Post-ZYMV	28.2 ± 1.76 c	17.1 ± 2.77 d	4.70 ± 0.26 c	0.43 ± 0.15 c	0.49 ± 0.11 c	0.13 ± 0.06 c	29.5 ± 2.25 d

Mock: plants inoculated with viral inoculation buffer and free-ISP-4 medium; ZYMV: plants mechanically inoculated with ZYMV; *S. fungicidicus*: plants treated with *S. fungicidicus-*culture filtrate (CF); Pre-ZYMV: plants treated with *S. fungicidicus*-CF, 24 h before ZYMV inoculation (protective); Post‐ZYMV: plants treated with culture filtrate of *S. fungicidicus*-CF, 24 h after ZYMV inoculation (curative). Each value represents the mean of five biological replicates. The mean values of each column with the same letter are not significantly different.

### Effect of *S. fungicidicus* on the peroxidase and total protein levels

The peroxidase (POD) enzyme activity was considerably increased in plants treated with protective treatment (4.42 mM/g FW) and curative treatment (3.96 mM/g FW) compared to those treated with ZYMV treatment and the mock treatment (3.19 and 2.77 mM/g FW, respectively) at 5 dpi (Table 4). The highest level of POD (6.21 mM/g FW) generation was seen in the protective treatment at 10 dpi. The level of POD was markedly elevated in the curative treatment group (5.64 mM/g FW) and slightly increased in *S. fungicidicus* treatment group (3.24 mM/g FW) compared to the control (2.85 mM/g FW). After considering all relevant aspects, the assessment of the POD enzyme suggests that applying *S. fungicidicus* to the leaves has a significant capacity to reduce oxidative stress resulting from a ZYMV infection, primarily through enzymatic mechanisms. For total protein content, the ZYMV showed a considerable increase, reaching a maximum value of 2.12 mg protein/g FW (Table 4). Squash plants treated with *S. fungicidicus* 24 h after ZYMV inoculation exhibited a greater amount of accumulated total soluble protein content compared to squash plants that were treated with *S. fungicidicus* 24 h before ZYMV inoculation (1.87 and 1.35 mg protein/g FW, respectively). At 10 dpi, plants that were treated with ZYMV had the highest total protein buildup (1.81 mg protein/g FW) compared to plants that were not treated and plants that were treated with other treatments (Table 4). Nevertheless, the total protein content was dramatically reduced by both protective and curative treatments, with concentrations of 1.45 and 1.16 mg protein/g FW, respectively (Table 4).

**Table 4:** Effect of ZYMV and *S. fungicidicus* treatments on peroxidase, total protein levels, oxidative stress markers (H_2_O_2_ and MDA), total phenolic content (TPC), and free radical scavenging activity (DPPH) on squash plants.

DPI	Treatment	Peroxidase (mM/g FW)	Total Protein (mg/g FW	H_2_O_2_ (µM/g FW)	MDA (nmole/g FW)	TPC (mg GAE/g)	DPPH (%)
5	Mock	2.77 ± 0.21 e	1.73 ± 0.16 c	16.6 ± 0.54 e	212.5 ± 55.4 e	69.9 ± 4.62 d	80.3 ± 1.47 e
	ZYMV	3.19 ± 0.23 c	2.12 ± 0.18 a	32.8 ± 4.95 a	275.8 ± 57.3 a	32.7 ± 3.66 e	87.1 ± 1.77 d
	*S. fungicidicus*	2.91 ± 0.25 d	1.23 ± 0.10 e	19.9 ± 1.64 d	217.4 ± 15.9 d	75.3 ± 5.85 c	93.3 ± 1.40 b
	Pre-ZYMV	4.42 ± 0.35 a	1.35 ± 0.18 d	22.8 ± 1.06 c	227.5 ± 35.7 c	89.4 ± 4.30 b	98.3 ± 0.95 a
	Post-ZYMV	3.96 ± 0.29 b	1.87 ± 0.06 b	29.3 ± 3.79 b	239.2 ± 30.8 b	143.6 ± 8.33 a	91.9 ± 1.88 c
10	Mock	2.85 ± 0.17 e	1.19 ± 0.04 c	27.4 ± 7.42 c	203.6 ± 41.2 e	66.6 ± 6.13 a	55.5 ± 0.98 d
	ZYMV	5.61 ± 0.41 c	1.81 ± 0.12 a	47.7 ± 1.47 a	377.4 ± 31.4 a	50.4 ± 8.89 c	64.9 ± 1.13 c
	*S. fungicidicus*	3.24 ± 0.42 d	1.56 ± 0.22 b	28.8 ± 7.52 d	251.8 ± 20.6 d	64.2 ± 7.21 b	45.2 ± 0.91 e
	Pre-ZYMV	6.21 ± 0.38 a	1.45 ± 0.09 b	36.9 ± 4.28 c	284.4 ± 21.4 c	47.7 ± 4.21 d	70.3 ± 0.34 a
	Post-ZYMV	5.64 ± 0.31 b	1.16 ± 0.17 c	41.4 ± 2.51 b	325.2 ± 20.9 b	44.3 ± 9.88 e	65.6 ± 1.67 b

Each value represents the mean of five biological replicates. The mean values of each column with the same letter are not significantly different. Mock: plants inoculated with viral inoculation buffer and ISP-4 medium of *S. fungicidicus*; ZYMV: plants mechanically inoculated with ZYMV; *S. fungicidicus*: plants treated with culture filtrate of *S. fungicidicus*; Pre-ZYMV: plants treated with culture filtrate of *S. fungicidicus*, 24 h before ZYMV inoculation (protective); Post‐ZYMV: plants treated with culture filtrate of *S. fungicidicus*, 24 h after ZYMV inoculation (curative).

### Effect of *S. fungicidicus* on the oxidative stress markers (H_2_O_2_ and MDA)

At 5 dpi, the ZYMV treatment showed the highest H_2_O_2_ level (32.8 µM/g FW) compared to the mock treatment (16.6 µM/g FW). The foliar application of *S. fungicidicus* significantly decreased the H_2_O_2_ level (Table 4). The protective and curative treatments reported 22.8 and 29.3 µM/g FW, respectively. In a similar vein, the MDA results at 5 dpi showed that the ZYMV treatment group had higher levels of lipid peroxidation (275.8 µM/g FW) than the mock treatment (212.5 µM/g FW). The Pre-ZYMV and Post-ZYMV treatments, on the other hand, had lower MDA levels than the ZYMV group (227.5 and 239.2 µM/g FW, respectively) (Table 4). At 10 dpi, the squash plants treated with ZYMV exhibited the highest levels of H_2_O_2_ and MDA, with concentrations of 47.7 and 377.4 µM/g FW, respectively. The treatment with *S. fungicidicus* demonstrated a significant reduction in the two stress markers (Table 4). The protective treatment reported 36.9 and 284.4 µM/g FW for H_2_O_2_ and MDA, respectively.

### Effect of *S. fungicidicus* on the total phenolic content and free radical scavenging activity

As shown in Table 4, the ZYMV-treated plants exhibited a considerable decrease in total phenolic content (32.7 mg/g DM) compared to the mock-treated plants (69.9 mg/g DM) at 5 dpi. On the other hand, *S. fungicidicus* significantly increased the TPC, especially in the protective treatment, which reported a value of 89.4 mg/g DM. This suggests that the timing of the treatment is vital for encouraging the production of these defense compounds. Similarly, at 10 dpi, the protective treatment reported the highest TPC content of 87.7 mg/g DM, followed by *S. fungicidicus* and curative treatments, with approximately 74 mg/g DM (Table 4). At 5 dpi, the protective treatment showed the highest level of DPPH with 98.3%, followed by the *S. fungicidicus* and curative treatments with 93.3% and 91.9%, respectively (Table 4). Furthermore, the ZYMV treatment exhibited a substantial increase in the DPPH level at 5 and 10 dpi, with percentages of 87.1% and 64.9%, respectively, in comparison to the mock treatment, which had percentages of 80.3% and 55.5%, respectively.

### Effect of *S. fungicidicus* on the transcriptional levels of defense-related genes

**Fig. 5:**
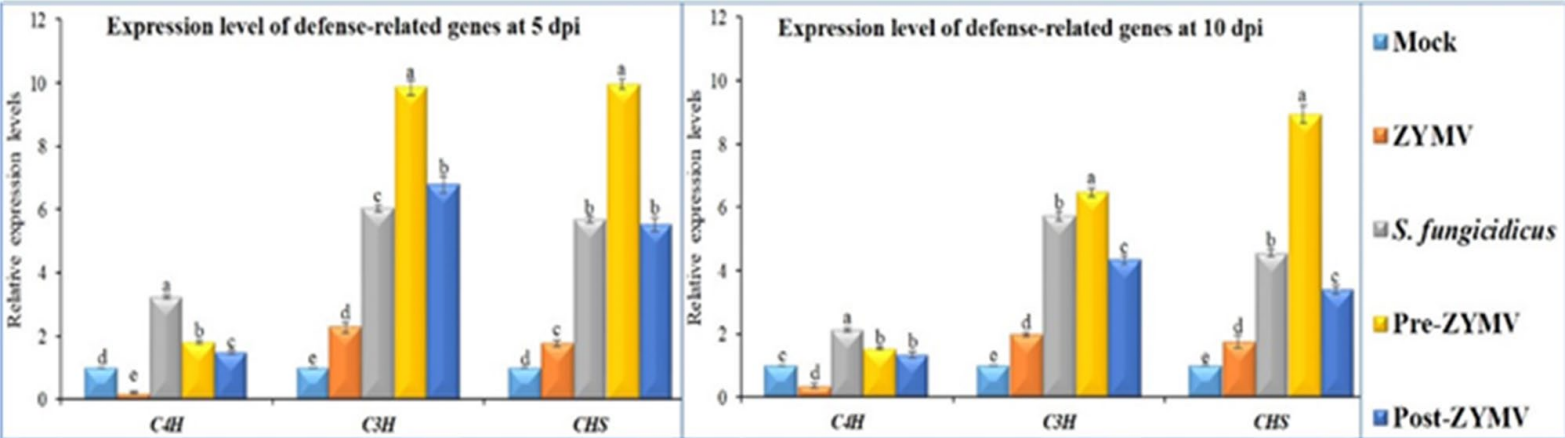
Relative expression levels of three polyphenolic genes (*C4H*, *C3H*, and *CHS*) in squash plants at 5 and 10 dpi. Mock: plants inoculated with viral inoculation buffer and ISP-4 medium of *S. fungicidicus*; ZYMV: plants mechanically inoculated with ZYMV; *S. fungicidicus*: plants treated with *S. fungicidicus*-CF; Pre-ZYMV: plants treated with *S. fungicidicus*-CF, 24 h before ZYMV inoculation; Post‐ZYMV: plants treated with *S. fungicidicus*-CF, 24 h after ZYMV inoculation. Each value represents the mean of five biological replicates. The values of each column with the same letter are not significantly different.

The relative expression levels of three polyphenol-related genes (*C4H*, *C3H*, and *CHS*) were evaluated at 5 and 10 dpi among the five different treatments (Fig. 5). At 5 and 10 dpi, the ZYMV treatment significantly decreased *C4H* expression, with levels 0.21- and 0.36-fold lower than those of the mock treatment (Fig. 5). The *S. fungicidicus* treatment reported the highest relative expression levels (3.22- and 2.14-fold), followed by protective (1.82- and 1.54-fold) and curative (1.49- and 1.33-fold) at 5 and 10 dpi, respectively. At 5 dpi, the RT-qPCR results for the *C3H* gene revealed a significant increase in its expression for both protective and curative treatments, with 9.85- and 6.78-fold increases compared to the control, respectively (Fig. 5). At 10 dpi, the relative expression levels were reported to be 6.46- and 4.35-fold higher than the control, respectively. The transcription level of ZYMV-treated plants was slightly increased, with values 2.27 and 1.99 times higher than the control at 5 and 10 dpi, respectively (Fig. 5). Similarly, the protective treatment exhibited the highest level of *CHS* relative expression at 5 and 10 dpi, with increases of 9.96 and 8.94 times that of the control (Fig. 5). On the other hand, the curative treatment increased *CHS* expression by 5.52 and 3.40-fold at 5 and 10 dpi, respectively. The ZYMV treatment induced moderate *CHS* expression, 1.77- and 1.74-fold at 5 and 10 dpi, respectively, which was higher than that of the mock treatment.

### Effect of *S. fungicidicus* on polyphenolic compounds

**Fig. 6:**
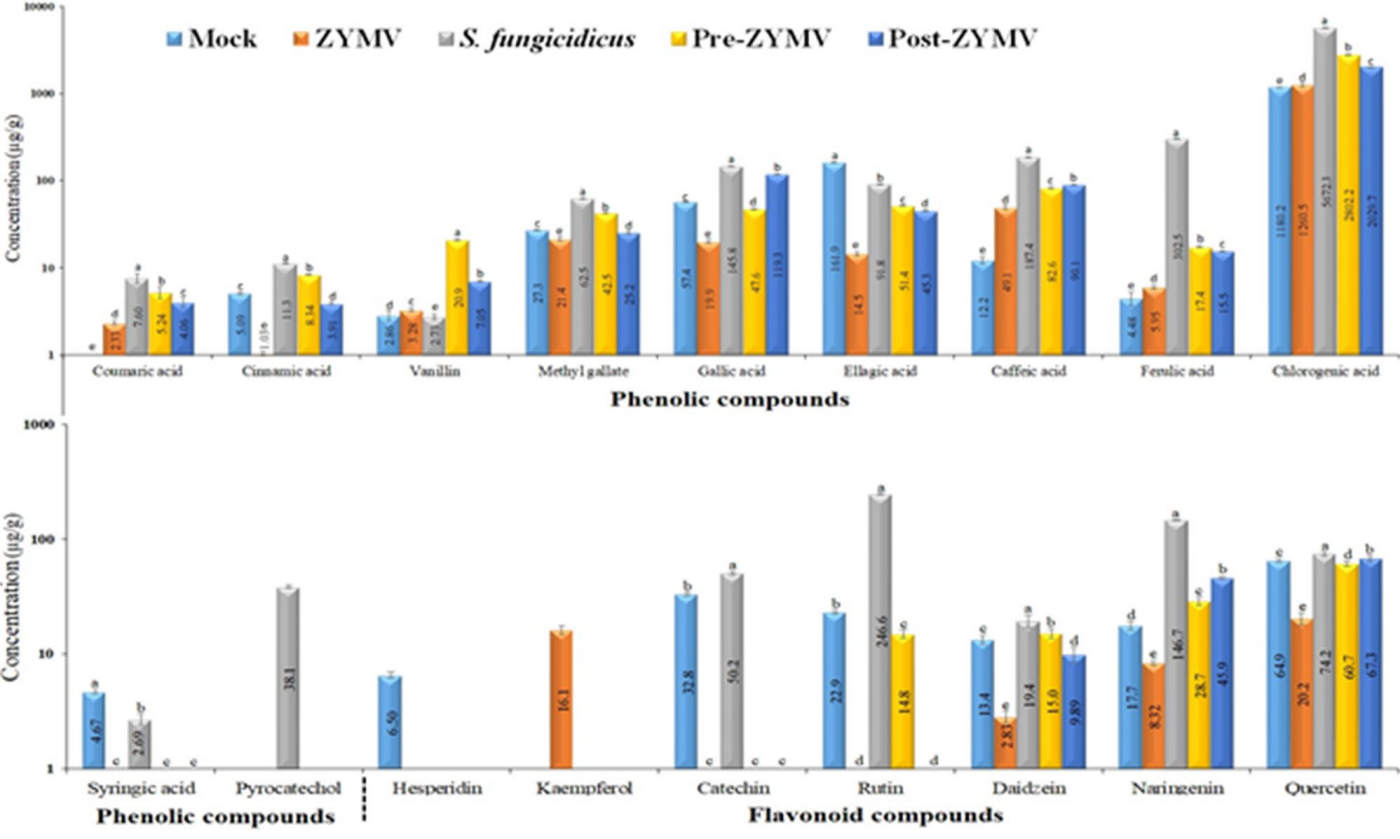
HPLC chromatograms of phenolic and flavonoid compounds identified in ethanolic squash extract at 10 dpi. Mock: plants inoculated with viral inoculation buffer and ISP-4 medium of *S. fungicidicus*; ZYMV: plants mechanically inoculated with ZYMV; *S. fungicidicus*: plants treated with *S. fungicidicus*-CF; Pre-ZYMV: plants treated with *S. fungicidicus*-CF, 24 h before ZYMV inoculation; Post‐ZYMV: plants treated with *S. fungicidicus*-CF, 24 h after ZYMV inoculation.

**Fig. 7:**
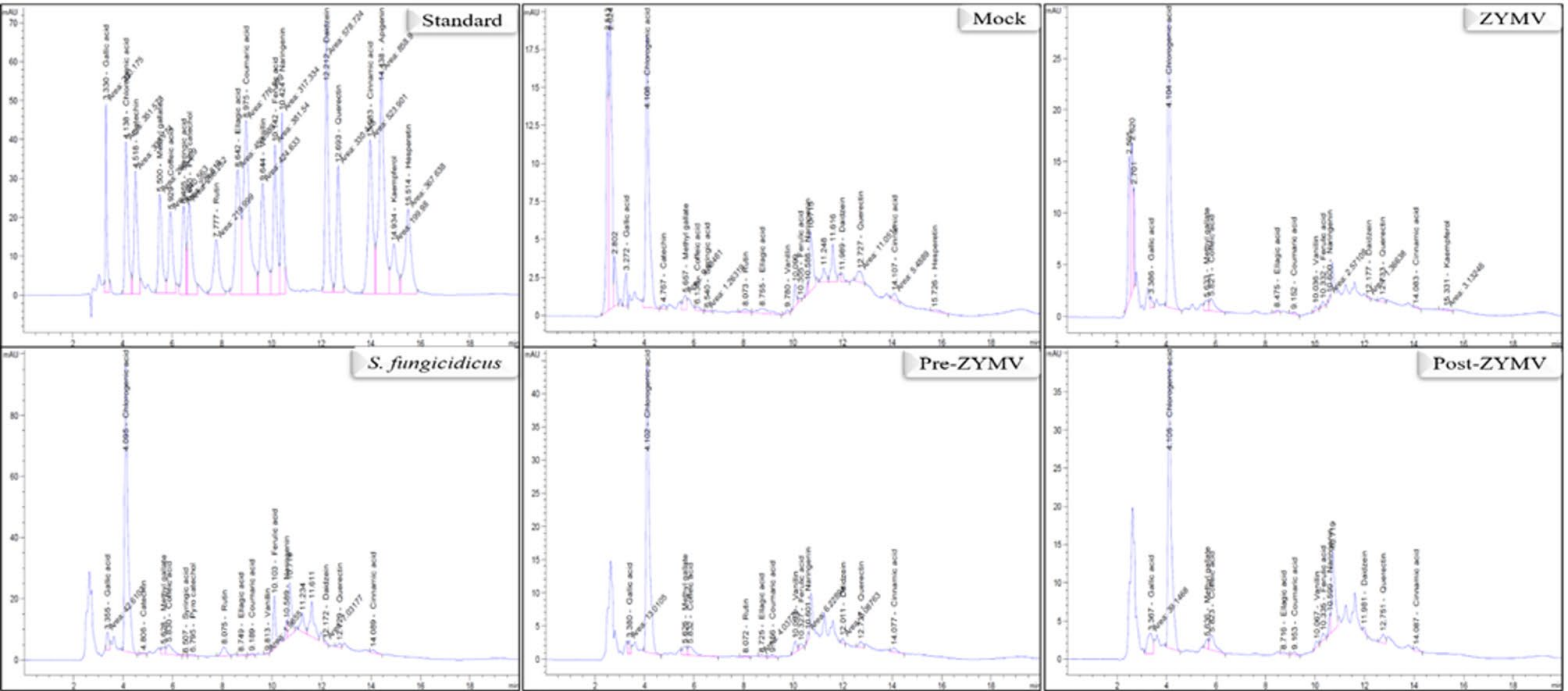
A histogram comparing the logarithmic relative accumulation levels of polyphenolic (phenolic and flavonoid) compounds detected in the ethanol extracts of squash leaves at 10 dpi under different treatments. Mock: plants inoculated with viral inoculation buffer and ISP-4 medium; ZYMV: plants mechanically inoculated with ZYMV; *S. fungicidicus*: plants treated with *S. fungicidicus*-CF; Pre-ZYMV: plants treated with *S. fungicidicus*-CF 24 h before ZYMV inoculation; Post-ZYMV: plants treated with *S. fungicidicus*-CF 24 h after ZYMV inoculation. Each value represents the mean of five biological replicates. Columns sharing the same letter are not significantly different from each other based on mean values.

HPLC chromatograms illustrated in Fig. 6 showed the detected flavonoid and phenolic compounds in squash plant leaves as affected by the foliar application of *S. fungicidicus* against the ZYMV experiment. The data presented in the bar chart (Fig. 7) illustrate the concentrations of each detected phenolic and flavonoid compound (in µg/g) in squash plants subjected to different treatments, including ZYMV, *S. fungicidus*, and their combinations (Pre-ZYMV and Post-ZYMV). The treatments are compared against a control group (Mock), and the data are displayed on a logarithmic scale, highlighting both moderate and significant variations in compound levels across treatments. Among the phenolic compounds (Fig. 7), chlorogenic acid stands out as the most abundant across all treatments, with the highest levels observed in the *S. fungicidus* (5672.3 µg/g) and Pre-ZYMV (2802.2 µg/g) groups. Other phenolic compounds, such as ellagic acid, gallic acid, and caffeic acid, also showed elevated levels in *S. fungicidus*-treated plants. In contrast, plants infected with ZYMV alone exhibited a significant reduction in the concentration of many phenolic compounds, including ferulic acid, vanillin, and syringic acid, indicating that the virus suppresses the plant’s phenolic metabolism. Interestingly, both Pre-ZYMV and Post-ZYMV treatments largely reversed these suppressive effects, often restoring or even surpassing the compound levels seen in the control group. In the case of flavonoid compounds (Fig. 7), rutin exhibited the highest concentration, particularly in the *S. fungicidus* treatment (246.6 µg/g), followed by mock treatment (22.9 µg/g), indicating a strong stimulatory effect of the *S. fungicidus* on flavonoid biosynthesis. Other flavonoids such as quercetin, catechin, and kaempferol were also significantly enhanced in *S. fungicidus*-treated plants. On the other hand, ZYMV treatment resulted in substantial reductions in many flavonoids, including naringenin, daidzein, and rutin, mirroring the trend observed with phenolic compounds. Once again, both Pre-ZYMV and especially Post-ZYMV treatments helped to restore or increase flavonoid levels, indicating the effectiveness of *S. fungicidus* as both a prophylactic and therapeutic agent. In summary, the data suggest that ZYMV negatively affects the biosynthesis of both phenolic and flavonoid compounds, potentially weakening the plant’s defense mechanisms. In contrast, treatment with *S. fungicidus* significantly enhances these compounds, even under viral stress. The Post-ZYMV treatment seems particularly effective, likely due to its curative action in countering the virus-induced suppression.

### Identification of bioactive metabolites of *S. fungicidicus*

**Fig. 8:**
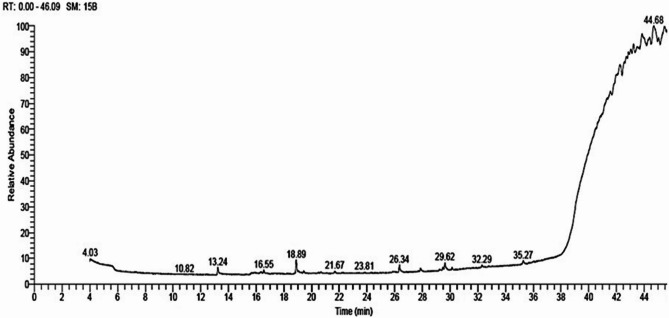
Histogram showing gas chromatography-mass spectrometry (GC-MS) fractionation of ethyl acetate extract of *Streptomyces fungicidicus* culture filtrate.

GC-MS equipment was used to identify the chemicals present in the culture filtrate of *S. fungicidicus* (Fig. 8). Table 5 presents each identified compound with its retention time (RT), area (%), molecular formula, and molecular weight. The ethyl acetate extract of *S. fungicidicus*-CF reveals the presence of 35 bioactive compounds. The most abundant compound was (-)-spathulenol, which had a peak area percentage of 13.13% at a retention time of 18.89 min. This was followed by 9-octadecenoic acid (z)-methyl ester, which had a peak area percentage of 9.24% at a retention time of 29.62 min, and triacetin, which had a peak area percentage of 8.88% at a retention time of 13.24 min (Table 5). Additional components exhibited different retention times and peaks.

**Table 5:** Composition of Ethyl acetate extract of *Streptomyces fungicidicus* isolate analyzed by GC-MS.

RT (min)	Compound name	Area %	Molecular formula	Molecular weight	Category
18.89	(-)-Spathulenol	13.13	C_15_H_24_O	220	Sesquiterpene
29.62	9-Octadecenoic acid (z)-, methyl ester	9.24	C_19_H_36_O_2_	296	FA methyl ester
13.24	Triacetin	8.88	C_9_H_14_O_6_	218	Triglyceride
26.34	Methyl-9,9,10,10-d4-octadecanoate	7.23	C_19_H_34_D_4_O_2_	302	FA methyl ester
4.03	Dotriacontane	6.60	C_32_H_66_	450	Alkane
35.28	4 H-1-benzopyran-4-one, 2-(3,4-dihydroxyphenyl)-6,8-Di-á-d-glucopyranosyl-5,7-dihydroxy-	4.85	C_27_H_30_O_16_	610	Flavonoid glycoside
27.86	Oxiraneoctanoic acid, 3-octyl-, cis-	3.67	C_18_H_34_O_3_	298	Epoxy fatty acid
16.55	3-Buten-2-one,4-(2,6,6-trimethyl-1-cyclohexen-1-yl)-	3.46	C_13_H_20_O	192	Terpenoid
5.62	1-Tetradecanol	3.30	C_14_H_30_O	214	Fatty alcohol
5.62	1-Hexadecanol, 2-methyl-	3.30	C_17_H_36_O	256	Alkaloid
32.29	Ethyl iso-allocholate	3.02	C_26_H_44_O_5_	436	Steroid
19.43	1,3,5-Triazine-2,4-diamine,6-chloro-n-ethyl-	2.68	C_5_H_8_ClN_5_	173	Triazine herbicide
29.48	9,12-Octadecadienoic acid (Z, Z)-,2-hydroxy-1-(hydroxymethyl)ethyl Ester	2.61	C_21_H_38_O_4_	354	Monoglyceride
21.67	Docosane	2.57	C_22_H_46_	310	Alkane
30.13	Cyclopropanedodecanoic acid, 2-octyl-, methyl ester	2.47	C_24_H_46_O_2_	366	Cyclopropane FA methyl ester
5.55	2-Hexadecanol	2.08	C_16_H_34_O	242	Fatty alcohol
20.67	2-Acetyl-3-(2-cinnamido)ethyl-7-methoxyindole	1.94	C_22_H_22_N_2_O_3_	362	Indole
15.62	9,10-Secocholesta-5,7,10(19)-triene-3,24,25-triol, (3á,5z,7e)-	1.94	C_27_H_44_O_3_	416	Sterols
36.02	Tetraneurin - a - Diol	1.84	C_15_H_20_O_5_	280	Terpene
29.23	Tetraneurin - a - Diol	1.84	C_15_H_20_O_5_	280	Terpene
32.78	Octadecanal, 2-bromo-	1.64	C_18_H_35_BrO	346	Bromoalkane
20.15	N-(2-{4,5-dimethoxy-2-[2-phenylethenyl]phenyl}-3-phenylpropyl)-n, n-dimethylamine hydrochloride	1.63	C_27_H_32_ClNO_2_	437	
25.87	1-Heptatriacotanol	1.62	C_37_H_76_O	536	Fatty alcohol
16.36	Isochiapin B	1.43	C_19_H_22_O_6_	346	Alkaloid
23.80	Dotriacontane	1.31	C_32_H_66_	450	Alkane
22.20	9-Oximino-2,7 diethoxyfluorene	1.23	C_17_H_17_NO_3_	283	Fluorene
20.49	Isochiapin B	1.21	C_19_H_26_O_6_	350	
15.93	2-Myristynoyl pantetheine	1.11	C_25_H_44_N_2_O_5_S	484	Vitamin
32.41	Glycidyl oleate	1.10	C_21_H_38_O_3_	338	Carboxylic ester
22.28	Limonen-6-ol, pivalate	0.91	C_15_H_24_O_2_	236	Terpenoid
36.80	4 H-1-benzopyran-4-one,2-(3,4-dimethoxyphenyl)-3,5-dihydroxy-7-methoxy-	0.86	C_18_H_16_O_7_	344	Flavonoid
35.67	12-Methyl-E, E-2,13-octadecadien-1-ol	0.75	C_19_H_36_O	280	Fatty alcohol
16.30	2,2,3,3,4,4 Hexadeuterooctadecanal	0.75	C_18_H_30_D_6_O	274	Isotopologue
27.67	9-Octadecenoic acid (z)-	0.68	C_18_H_34_O_2_	282	Unsaturated FA
18.24	Morphinan-4,5-epoxy-3,6-di-ol,6-[7-nitrobenzofurazan-4 yl]amino-	0.64	C_26_H_27_N_5_O_6_	505	Morphinan
35.97	9,12,15-Octadecatrienoic acid, 2,3-bis[(trimethylsilyl)oxy] propyl ester, (z, z,z)-	0.36	C_27_H_52_O_4_Si_2_	496	Fatty acid

## Discussion

Globally, economically essential crops, including wheat, rice, potatoes, soybeans, squash, and maize, are frequently affected by severe diseases caused by various plant pathogens, such as bacteria, viruses, nematodes, and fungi^55,56^. Among these, ZYMV, a single-stranded RNA virus, is one of the most destructive, causing up to 100% yield loss when infection occurs before flowering, often accompanied by severe physiological and morphological abnormalities in plants^7,57^. In this study, the Egyptian isolate of ZYMV (RZ24) was successfully isolated, purified, and molecularly characterized using the CP gene, and subsequently deposited in GenBank, ensuring its accessibility for future research. The phylogenetic tree of the RZ24 isolate was positioned within a well-supported clade alongside geographically and temporally diverse ZYMV isolates, based on the phylogeny of the CP gene. It clustered with other Egyptian isolates (e.g., MN422073), Syrian (e.g., MK606175), and broader Group A isolates that include sequences from the USA, suggesting its affiliation with a widely distributed and established lineage of ZYMV^58^. Globally, CP-based phylogenetic studies delineate ZYMV isolates into two major clades, with Group I comprising largely singletons (e.g., the Chinese variant) and Group II encompassing the majority of isolates from Asia, Europe, the Americas, and the Middle East^59^. Within this context, Egyptian isolates like RZ24 fall within subclade IIa, reflecting their international linkage yet hinting at localized diversification^59^. The variation observed among ZYMV isolates can often be traced to geographic and ecological factors, as demonstrated in European isolates (Austria, Slovenia, Germany), where local clusters correspond well to regional proximity^60^. Moreover, in regions like Iran and Iraq, high genetic similarity (> 98% identity) between local isolates and American strains indicates frequent exchange or shared evolutionary history^61^. Isolate RZ24 thus represents an Egyptian strain that, while belonging to the widespread Group A, exhibits moderate divergence from other global isolates—likely reflecting regional adaptation and local genetic drift. Its placement within Group A underscores both its potential to contribute to global ZYMV epidemiology and its value in informing region-specific management strategies. Therefore, managing such pervasive plant viruses, e.g., ZYMV, is a primary priority for improving food security and ensuring agricultural sustainability. *Actinobacteria* are considered potential biocontrol agents for plant diseases, and they are an effective alternative to chemical pesticides in managing plant viral diseases^22^. Their activity is attributed to mechanisms such as siderophore production, hydrolytic enzymes (e.g., chitinases and glucanases), antibiotics, and hydrogen cyanide^62,63^, as well as their ability to enhance plant growth, induce systemic resistance, and delay symptoms.

Under greenhouse conditions, the application of *Streptomyces fungicidicus* culture filtrate (*S. fungicidicus*-CF) as both a protective and curative treatment resulted in significant improvements in growth parameters, chlorophyll content, and a substantial reduction in viral accumulation and symptom appearance. Viral accumulation decreased by 49.7% at 5 dpi and 37.1% at 10 dpi in protective treatments, indicating its promising antiviral activity. These findings align with previous reports, such as those by Abdelkhalek et al.^30^, where *Paenibacillus polymyxa* culture filtrate suppressed ZYMV infection, and Abo-Zaid et al.^22^, who demonstrated that *Streptomyces cellulosae* enhanced tomato growth under TMV challenge. The ability of *S. fungicidicus* to promote plant growth may be attributed to its capacity to enhance the production of various plant growth hormones. This finding is in agreement with the study by Ghanem et al.^20^ who reported that a substance present in the culture filtrate of *Streptomyces* spp. not only inhibited ZYMV replication but also promoted plant growth and induced systemic resistance in host plants. This actinobacterium synthesizes a range of phytohormones, including auxins, cytokinins, and gibberellins, which collectively stimulate cell division, cell elongation, and the expansion of root surface area by promoting the development of adventitious and lateral roots^64^. In the current study, the protective treatment of squash plants with *S. fungicidicus*-CF led to a marked reduction in ZYMV accumulation levels compared to untreated infected plants. These reductions can be attributed to the presence of biologically active metabolites in the culture filtrate, which not only inhibit viral replication but also enhance the innate defense mechanisms, thereby conferring a dual protective effect^25,65^.

Peroxidase (POD) is a pivotal enzyme in the antioxidant defense system of plants, where it neutralizes reactive oxygen species (ROS) generated during biotic and abiotic stresses, thereby preventing oxidative damage to cellular structures^66,67^. In the current study, foliar application of *S. fungicidicus*-CF markedly increased POD activity in both protective and curative treatments at two different time points post-inoculation. This observation supports earlier findings by Li et al.^68^, who reported that *S. pactum* Act12 enhanced peroxidase activity in tomato plants infected with tomato yellow leaf curl virus (TYLCV). Similarly, *S. chromofuscus* was found to induce peroxidase activity in TYLCV-infected tomato plants^21^. Comparable findings have been reported for other actinobacterial strains, where *Streptomyces* inoculation activated peroxidase and other defense-related enzymes in potato, wheat, and eucalyptus, enhancing systemic resistance against viral, bacterial, and fungal pathogens^69–71^. These results indicate that actinobacteria, particularly *Streptomyces*, not only act as biocontrol agents but also function as potent elicitors of the plant’s antioxidant machinery^72,73^. The enhanced POD activity observed in our study suggests that *S. fungicidicus*-CF effectively primed the antioxidant defense system of squash plants, mitigating oxidative damage typically associated with ZYMV infection. This aligns with previous reports showing that actinobacterial metabolites can activate systemic resistance pathways through salicylic acid (SA) and jasmonic acid (JA)-dependent signaling^69,72,73^. Such priming not only enhances the plant’s capacity to detoxify ROS but also strengthens structural and biochemical defenses, improving resilience to viral stress. Regarding total soluble protein content, our results demonstrated that ZYMV-infected squash plants exhibited the highest protein accumulation at 10 dpi. This increase may be attributed to the synthesis of viral coat proteins, accumulation of stress-related proteins, and the induction of host defense proteins during infection. Interestingly, no significant differences in total protein content were observed between protective and curative treatments with *S. fungicidicus*, suggesting that while the bacterium enhanced enzymatic defense responses, its effect on protein accumulation was less pronounced at this stage of infection. Similar elevations in soluble protein content have been reported in faba bean plants infected with bean yellow mosaic virus^67,74^, while other studies documented a reduction in total soluble protein under viral stress due to the degradation of host proteins and suppression of metabolic pathways^30,75^. These contrasting trends highlight that the response of protein metabolism to viral infection is complex and may vary depending on the host species, viral strain, and timing of the analysis.

Lipid peroxidation-induced membrane rupture and damage to plant cells are evident from the significant increase in reactive oxygen species resulting from stress conditions and the generation of MDA^76,77^ The two oxidative stress indicators, H_2_O_2_ and MDA, were significantly elevated and reached their maximum levels in the ZYMV-treated plants. Interestingly, protective and curative treatments showed a noteworthy decrease in both oxidative stress markers compared to the viral treatment. The findings were comparable to previous reports that showed viral infection induced MDA and H_2_O_2_ in infected plants^41,78,79^. Compared to ZYMV treatment, the curative treatment revealed a substantial rise in the total phenolic content. There were appreciable differences in the TPC among the squash plants treated with *S. fungicidicus*-CF, Pre-ZYMV, and Mock treatments, especially at 5 dpi. Phenolic comounds are essential to plants’ defensive mechanisms, as they enable them to withstand viral infections and reduce their vulnerability to oxidative stress^80,81^. It’s interesting to note that DPPH levels vary significantly across all treatments.

According to several research data, *p*-coumaroyl CoA is converted to naringenin chalcones by *CHS*, the first essential enzyme in flavonoid biosynthesis in several plants^82,83^. *CHS* is necessary for the formation of flavonoids in a variety of plant tissues^84^. The squash plants treated with *S. fungicidicus*-CF, ZYMV, protective and curative treatments showed up-regulated transcripts of *CHS* at 5 and 10 dpi compared to the Mock plants. At 5 and 10 dpi, the protective treatments were found to have the highest transcriptional levels of *CHS*, with relative expression levels higher than those of the Mock plants. Thus, we propose that *S. fungicidicus* might function as an elicitor molecule, inducing the immunological defense system and perhaps leading to SAR activation. In a recent study, Actino-48-treated tomato plants exhibited an increase in *CHS* activity in their leaves, resulting in the formation of SAR against TMV^22^. In our research, *C4H* levels at 5 and 10 dpi decreased in response to ZYMV treatment, with relative expression levels indicating a decrease compared to the Mock treatment. At 5 and 10 dpi, the *C4H* expression was most significant in the *S. fungicidicus*, with a fold change in relative transcriptional level. The protective and curative treatments demonstrated a considerable fold-increase in *C3H* activity at 5 dpi compared to the Mock plants.

To understand how biological control functions in novel applications, it is crucial to monitor the secondary metabolites generated by microorganisms using a variety of analytical methods. These compounds act as precursors for a broad range of biological processes^85^. The current investigation identified the bioactive components in an ethyl acetate extract of *S. fungicidicus*-CF using GC-MS equipment. GC-MS analysis indicates that *S. fungicidicus*-CF is composed of 35 distinct components with the most potent concentration of (-)-spathulenol, followed by triacetin, methyl ester, and 9-octadecenoic acid (z)-^86^. Describe dotriacontane as a long-chain alkane molecule with many biological actions, such as antibacterial and antioxidant capabilities^87^. It was found that the filtrate of *Streptomyces capoamus* contains dotriacontane, which inhibits *Ralstonia solanacearum*, the bacterium that causes wilt disease on banana plants. An alkaloid molecule called 1-hexadecanol, 2-methyl-was found in *Ricinus communis* leaf ethanol extract, together with other alkaloid compounds. These phytocompounds were shown to possess antioxidant and antibacterial properties^88^. According to Tan et al.^89^, *Salvia cilicica* oil contains other common compounds, such as (-)-spathulenol, which is thought to be the predominant component (23.8%) and exhibits antimycobacterial activity against *Mycobacterium tuberculosis*, *Microsporum gypseum*, *Trichophyton mentagrophytes*, and *Candida* spp. Moreover, immunomodulatory, antiproliferative, and anti-inflammatory properties. Additionally, isochiapin B exhibited anticancer, antioxidant, and antimicrobial properties. Other substances possess antibacterial actions, such as docosane^90^. Additionally, 9-octadecenoic acid (z)-,methyl ester was shown to have antibacterial action in the filtrate of *Streptomyces capoamus*^87^. The diversity of metabolites in *S. fungicidicus*-CF suggests a synergistic mechanism that integrates direct antiviral effects with enhanced plant defense responses. Collectively, these findings establish *S. fungicidicus* as a potent biocontrol agent against ZYMV, providing dual benefits of viral suppression and plant growth promotion. To precisely identify the mechanism or mechanisms by which *S. fungiciducus* inhibits plant viruses such as ZYMV, additional investigation is necessary.

## Conclusions

In conclusion, this study demonstrates the significant potential of *Streptomyces fungicidicus* as a biocontrol agent against ZYMV in squash crops. The effective isolation and characterization of ZYMV, combined with the application of *S. fungicidicus*, revealed notable improvements in managing ZYMV infection. The pre-inoculation treatment with *S. fungicidicus* not only delayed the onset of symptoms but also substantially reduced viral accumulation and enhanced plant growth, chlorophyll content, and defense responses. The ability of *S. fungicidicus* to increase peroxidase activity, total phenolic content, and the expression of key defense-related genes underscores its role in boosting plant resistance. Moreover, the identification of bioactive compounds, such as (-)-spathulenol, further supports its potential as an effective and eco-friendly solution for managing viral infections in squash. Overall, *S. fungicidicus* offers a promising alternative to chemical pesticides, contributing to more sustainable agricultural practices.

## Data Availability

The sequencing datasets generated and analysed during the current study have been deposited at the NCBI database under the GenBank accession numbers (PV489988 and PV131044) that are publicly accessible at https://www.ncbi.nlm.nih.gov. The data that supports the findings is included in the publication or is available from the corresponding author upon reasonable request.
